# Self-Gated CINE MRI for Combined Contrast-Enhanced Imaging and Wall-Stiffness Measurements of Murine Aortic Atherosclerotic Lesions

**DOI:** 10.1371/journal.pone.0057299

**Published:** 2013-03-05

**Authors:** Brigit den Adel, Linda M. van der Graaf, Gustav J. Strijkers, Hildo J. Lamb, Robert E. Poelmann, Louise van der Weerd

**Affiliations:** 1 Anatomy & Embryology, Leiden University Medical Center, Leiden, The Netherlands; 2 Biomedical NMR, Department of Biomedical Engineering, Eindhoven University of Technology, Eindhoven, The Netherlands; 3 Radiology, Leiden University Medical Center, Leiden, The Netherlands; 4 Human Genetics, Leiden University Medical Center, Leiden, The Netherlands; Leiden University Medical Center, The Netherlands

## Abstract

**Background:**

High-resolution contrast-enhanced imaging of the murine atherosclerotic vessel wall is difficult due to unpredictable flow artifacts, motion of the thin artery wall and problems with flow suppression in the presence of a circulating contrast agent.

**Methods and Results:**

We applied a 2D-FLASH retrospective-gated CINE MRI method at 9.4T to characterize atherosclerotic plaques and vessel wall distensibility in the aortic arch of aged ApoE^−/−^ mice after injection of a contrast agent. The method enabled detection of contrast enhancement in atherosclerotic plaques in the aortic arch after I.V. injection of micelles and iron oxides resulting in reproducible plaque enhancement. Both contrast agents were taken up in the plaque, which was confirmed by histology. Additionally, the retrospective-gated CINE method provided images of the aortic wall throughout the cardiac cycle, from which the vessel wall distensibility could be calculated. Reduction in plaque size by statin treatment resulted in lower contrast enhancement and reduced wall stiffness.

**Conclusions:**

The retrospective-gated CINE MRI provides a robust and simple way to detect and quantify contrast enhancement in atherosclerotic plaques in the aortic wall of ApoE^−/−^ mice. From the same scan, plaque-related changes in stiffness of the aortic wall can be determined. In this mouse model, a correlation between vessel wall stiffness and atherosclerotic lesions was found.

## Introduction

Cardiovascular diseases, in particular carotid and other peripheral atherosclerotic diseases are the leading causes of death in the western world [1]. Remodeling of the arterial wall intima, media and adventitia layers leads to the formation of an atherosclerotic plaque that may over time progress towards a vulnerable, rupture-prone phenotype [2]. Rupture of a plaque and subsequent myocardial infarction or stroke accounts for more than 50% of all cardiovascular deaths [1].

Clinical predictors for cardiovascular events due to vulnerable plaque rupture are plaque components like intraplaque macrophage content and the extent of the lipid core. Apart from the composition of atherosclerotic plaques, arterial stiffness and distensibility are independent predictor of cardiac morbidity. Despite this independency, atherosclerotic plaques do contribute significantly to the vessel wall stiffness, and changes in plaque burden or aortic compliance could help to identify early cardiovascular disease in patients before an actual plaque rupture, as well as monitor the results of the therapeutic interventions [3], [4]. Hydroxy-3-methylglutaryl coenzyme A reductase inhibitors, or statins, are well known to exert beneficial effects on the elastic properties of the arterial wall [5]. They are widely applied in both the clinic as well as in preclinical studies.

Much effort has been put into development of non-invasive techniques such as MRI to image the presence of atherosclerotic plaque directly using (targeted) contrast agents [6]–[10]. Separately, MRI techniques have been employed to image arterial stiffness, also called vascular compliance [11]–[16] and distensibility through cyclic strain calculations.

In this report we describe the simultaneous determination of plaque burden in the aortic arch and the stiffness and distensibility of the vessel wall of mice using retrospective-gated CINE MRI.

Retrospective-gating provides a method to depict both contrast agent enhancement in the atherosclerotic plaque at atheroprone vessels, such as the ascending aorta, which are characterized by motion due to the beating heart as well as oscillatory flow. This self-gated navigator-based CINE MRI technique is nowadays widely applied for cardiac MRI, allowing continuous acquisition of data points without the need for respiratory and ECG sensors [17]. The technique is based on the acquisition of a navigator signal with every k-space line, followed by sorting data points according to their origin in the cardiac and respiratory cycle [18]. As the vessel wall images are reconstructed separately for different phases in the cardiac cycle, CINE movies can be created of vascular diameter, from which the vascular compliance can be determined.

In this study, we used the ApoE^−/−^ mouse model that spontaneously develops atherosclerotic lesions of morphology similar to that observed in humans [19]–[21] to investigate the value of retrospective-gated CINE MRI of the aortic arch for atherosclerotic plaque detection and assessment of wall stiffness after injection of contrast agents that home to macrophages [9], [22], [23]. A common consequence of atherosclerosis, observed both in humans and in animal models, is an increase in the stiffness of the aorta and major arteries, resulting in decreased vascular elasticity and compliance [13], [24]. Therefore we assessed whether we could determine the stiffness of the aortic arch based on the self-gated MRI data.

Retrospective-gated MRI was done in young (12 weeks) and aged (12–14 months) ApoE^−/−^ mice with advanced atherosclerotic plaques in the bases of the aortic arch, assessing the presence of atherosclerotic plaques and vascular compliance as a function of disease progression, as well as during a therapeutic intervention with atorvastatin.

## Materials and Methods

### *In vivo* Experiments

All experiments were conducted in accordance with the Dutch guidelines for research animal care. Two groups (n = 5 per group) of 12 weeks old male ApoE^−/−^ mice on a C57BL/6/Jico background were fed a normal chow diet. Four groups of 12-to-14-month-old mice (n = 5 per group) were either fed (n = 2 groups) a Western high fat diet (1% cholesterol, Ab Diets) or a (n = 2 groups) Western diet supplemented with 0.01% wt/wt atorvastatin (Lipitor, Pfizer) ( = 0.1 g statin/kg bodyweight) for 12 weeks.

Plaque imaging was performed with contrast enhancement using either Gd-containing micelles or ultra-small iron oxide particles. Optimal time points for contrast agent accumulation in the plaque were determined in a pilot time-course study with n = 8 mice per group in which contrast agent accumulation was followed over time for 7 days with intervals ranging from 30 minutes to 6 hours (Figure S1). The optimal time point was defined as the time at which a stable amount of contrast enhancement (contrast-to-noise ratio (see below) significantly increased compared to the unenhanced aortic arch) and relatively small standard deviations due to between-animal variations was observed. The optimal imaging time was determined to be 12 h p.i. for the Gd-micelles [25], and 24 h for the USPIO particles. The blood circulation half-time of the micelles in the circulation were determined by fitting the ΔR1 and ΔR2 values using a mono-exponential decay function. The half-time is 8.3 hours and the half-time of the USPIOs is 10.4 hours.

Each mouse was scanned before administration of contrast agent and at the optimal time point after intravenous injection of micelles equivalent to 50 µmol Gd^3+^-DTPA lipid/kg bodyweight diluted in 200 µl, or 250 µmol Fe/kg bodyweight USPIO in 200 µl dextrose solution. Mice were anesthetized with isoflurane (2% in 1∶1 oxygen:air). During the examination, the respiration rate was continuously measured with a balloon pressure sensor connected to the ECG/respiratory unit. The isoflurane concentration was adjusted to keep the respiration rate between 50 and 90 respirations/min. Rectal temperature during the experiment was 35^o^C.

### Contrast Agents

#### Gadolinium-Based T1 Contrast Agent

Micelles were prepared by lipid film hydration [26]. A mixture of the appropriate amounts of lipids (typically 120 mol of total lipid) was dissolved in chloroform/methanol 3∶1 (v/v) and evaporated to dryness by rotary evaporation at 37°C. Gd-DTPA-BSA (Gd-DTPA-bis(stearylamide), PEG2000-DSPE (1,2-distearoyl-sn-glycero-3-phosphoethanolamine-N-[methoxy(polyethyleneglycol)-2000]) (all Avanti Polar lipids Inc) were used at a molar ratio of 1.5/1.35. For fluorescent detection, 0.1 mol% NIR664-DSPE (SyMO-CHEM B.V., Eindhoven, The Netherlands) was added. The lipid film was subsequently hydrated in HEPES buffered saline (HBS), containing 20 mM HEPES and 135 mM NaCl (pH 7.4) and vigorously stirred at 65°C for 45 min. The size and size distribution of the micelles were determined by dynamic light scattering (DLS) at 25°C with a Malvern 4700 system (Malvern ZetaSizer Nano S, Malvern, UK). The micelles had a mean size of 16 nm and a polydispersity index below 0.1, which indicates a narrow size distribution. The relaxivity was measured at 37°C and 9.4 T. The phospholipid content of the liposome preparations was determined by phosphate analysis according to Rouser after destruction with perchloric acid [27].

Iron-oxide-based T2* contrast agent: ultra-small superparamagnetic iron oxides (USPIO, Sinerem®) were obtained from Guerbet (Guerbet group, Aulnay sous Bois, France). An equivalent of 250 µmol Fe/kg bodyweight was injected i.v.

### MRI Protocols

All experiments were performed with a vertical 89-mm bore 9.4 T magnet (Bruker, Ettlingen, Germany) supplied with an actively shielded Micro2.5 gradient system of 1 T/m and a 30 mm transmit/receive birdcage RF coil, using Paravision 4.0 software.

At the start of each examination, several 2D Fast Low Angle Shot (FLASH) scout images were recorded in the transverse and axial plane of the heart to determine the orientation of the aortic arch. A modified 2D FLASH sequence with a navigator echo (IntraGate, Bruker) was used for retrospective CINE MRI with the following parameters: Hermite-shaped RF pulse 1 ms; FA 15°; TR 31.4 ms; TE 2.96 ms; navigator echo points 64; 10 cardiac frames; FOV 1.8*1.8 cm^2^; matrix 128*96, zero-filled to 128*128; in-plane resolution 141 µm; 6 concomitant slices covering the inner curvature of the aortic arch; slice thickness 0.4 mm; number of repetitions 400; total acquisition time approximately 20 min. Images were positioned both perpendicular to and in line with the aortic arch according to an external placed reference to assure maintenance of the positioning plane pre and post contrast agent injection.

We performed aortic diameter measurements with 5, 8, 12, 15, 20 and 40 frames to assess the variability in the diameter measurements in a group of 3 months old (hemodynamically stable) ApoE^−/−^ mice (n = 5) in relation to the frame number. All further analysis were performed using 10 reconstructed frames (Figure S2).

### Image Analysis

**Figure 1:**
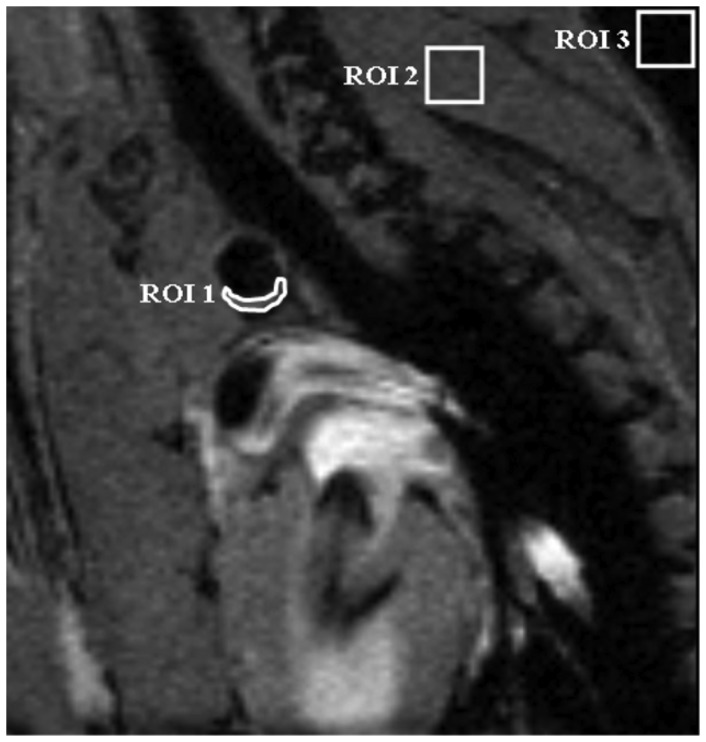
Contrast-to-Noise determination in the aortic arch. Region of interest (ROI) placement in the MR images were used to determine the contrast to noise ratios (CNR) in the atherosclerotic plaques in black blood images of a cross section of the aortic arch. ROI1 is placed in the atherosclerotic plaque in the vessel wall (Iwall). ROI2 is positioned in a muscle and used for normalization purposes (Imuscle). Noise levels were determined in ROI 3, placed in a region without signal. The standard deviation of the noise (stdevnoise) was used for normalization purposes.

Images were analyzed using ImageJ software. For contrast to noise determination of micelles, black blood images in 3 to 4 adjacent cross-sectional slices (the ones that had the lowest signal intensity, i.e. black-blood) through the aortic arch were analyzed (Figure 1). For USPIO the bright blood images were analyzed. ROIs were semi-automatically drawn around the vessel wall (I_wall_) in all 10 movie frames. A 2^nd^ ROI was drawn in the surrounding muscle tissue of the shoulder girdle (I_muscle_). Furthermore, an ROI was placed outside the animal to measure the noise level (SD_noise_). The contrast–to-noise ratio was defined in the 3 to 4 adjacent movie frames with the lowest signal intensity in the vessel lumen as follows:
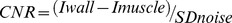
(1)


CNR values are presented as mean ± standard deviation.

To calculate the vessel wall stiffness, the cross-sectional diameter and area of the aortic arch were segmented manually in each frame. MRI slices were positioned orthogonal to the aortic arch, the frames that were obtained just before and after the branch of the left carotid artery had a stable circular shape and were used for this analysis (Figure S3).

For the determination of the circumferential strain as a measure of distensibility we assumed that 1) the deformation through the thickness of the vessel and 2) the deformation in the axial direction was small compared to the circumferential deformation, as previously described by Morrison *et al.* [28]. Assuming a circular cross section of the aorta, the following expression was used to calculate the circumferential cyclic strain,
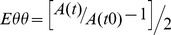
(2)where A is the cross sectional area of the aortic arch [15].

### Histology

Aortic arches were frozen in Tissue Tek® (Sakura Finetek Europe, Zoetermeer, The Netherlands) and cut into serial 5-µm sections. Sections were stained with hematoxylin and eosin for gross morphology and Oil Red O for lipid deposition as described previously [29], followed by bright-field microscopy. Lesion size was calculated from 8–10 consecutive H&E stained sections of the aortic arch. Iron deposits were visualized using Perl’s Prussian blue staining. Presence of Gd-containing micelles was indirectly detected by staining of the DTPA chelate present in the micelles as recently described in den Adel *et al.* [30]. In short, sections were incubated overnight at room temperature with a rabbit polyclonal primary antibody against Gd-DTPA (1∶20, BioPAL Inc). Goat-anti-rabbit conjugate (1∶200, DAKO) with normal goat serum diluted in PBS was incubated for 1 h at room temperature as secondary antibody. Biotin labelling was followed by development using black alkaline phosphatase (Vector Laboratories Inc., United Kingdom) and counterstaining was done with Mayer’s hematoxylin.

### Statistical Analysis

Data are represented as the mean ± standard deviation. Statistical analyses were performed using SPSS 17.0.2 (SPSS, Inc., Chicago, IL, USA). Statistical significance between groups was assessed using (paired) t-test, one-way analysis of variance (ANOVA), followed by a Bonferroni correction for multiple testing in case of significance. Results were considered statistically significant at p<0.05.

## Results

### Self-Gated CINE MRI in Cardiovascular Unstable Animals

**Figure 2:**
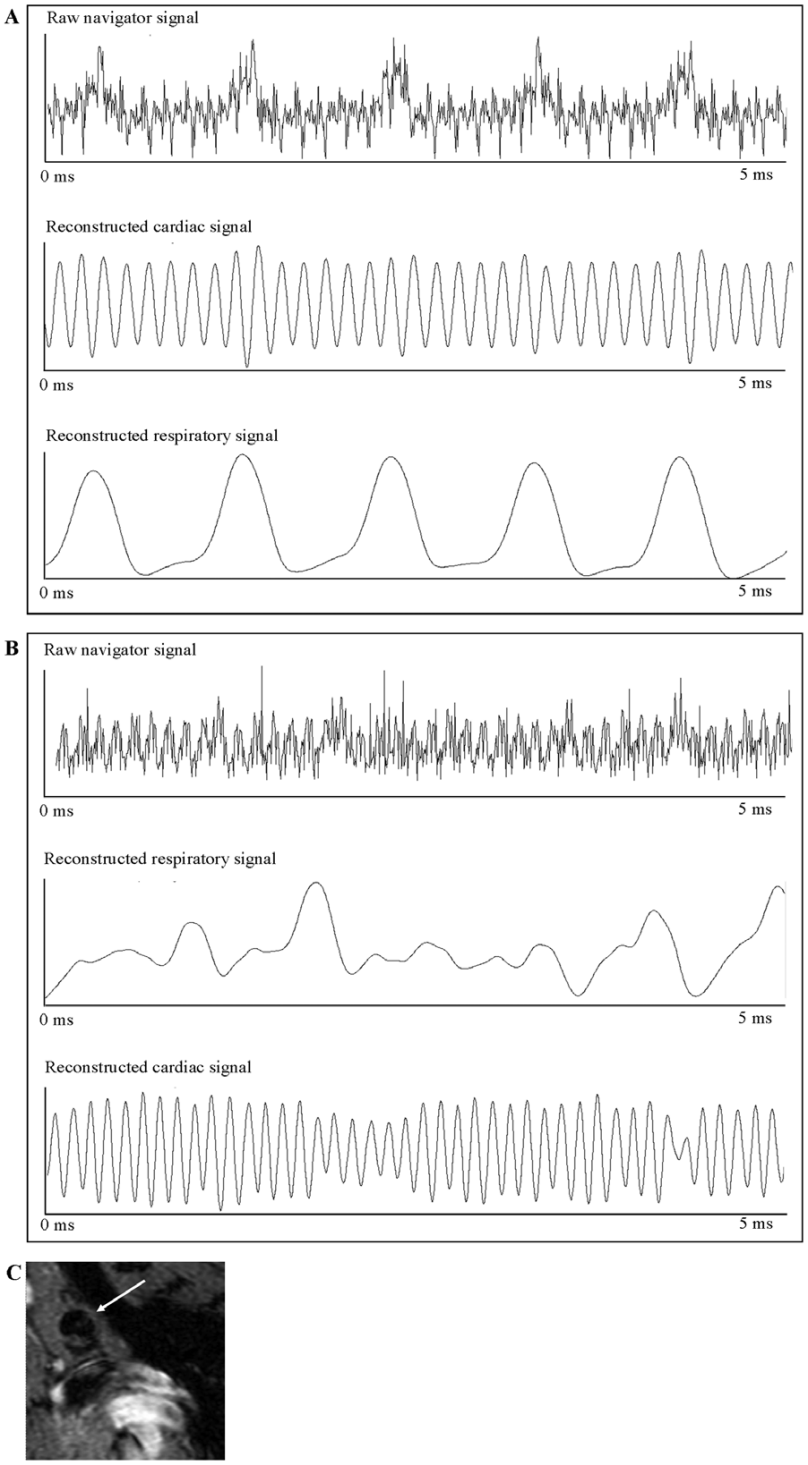
Navigator signals for the reconstruction of self-gated MRI. Analysis of navigator signals for reconstruction of the self-gated MR acquisitions. A. Example of a raw navigator signal with corresponding filtered respiratory and cardiac signals. B. Under unstable physiological situations it is still possible to gather correct cardiac and respiratory traces. With the filtered reconstruction signals of both, it is possible to re-order data points in such a way a clear image of the aortic arch can still be generated. C. Representative black blood image before injection of contrast agent from the same animal shown above, with impaired cardiac and respiratory function. Retrospective gating led to a stable reconstruction of the aortic arch (arrow).

All mice included in these experiments had a relatively low variation in heart rate and respiratory rate throughout the examinations, ranging from 490 to 520 beats/min and from 50 to 80 respirations/min respectively (data not shown). The navigator echo in this sequence was used to demerge a cardiac and respiratory signal and subsequently reconstruct the sample point according to the cardiac cycle (Figure 2A). However, even in cardiac and respiratory unstable mice it was feasible to obtain artifact-free MR images by specifically selecting the cardiac and respiratory weighting and periods used (Figure 2B). With retrospective-gated CINE MRI, the image reconstruction could be optimized after sampling all the data points; while maintaining the usual scan time, we could still generate correct and stable images of the aortic arch allowing for plaque detection and vessel wall delineation (Figure 2C).

### Imaging of Contrast Agent Uptake

**Figure 3:**
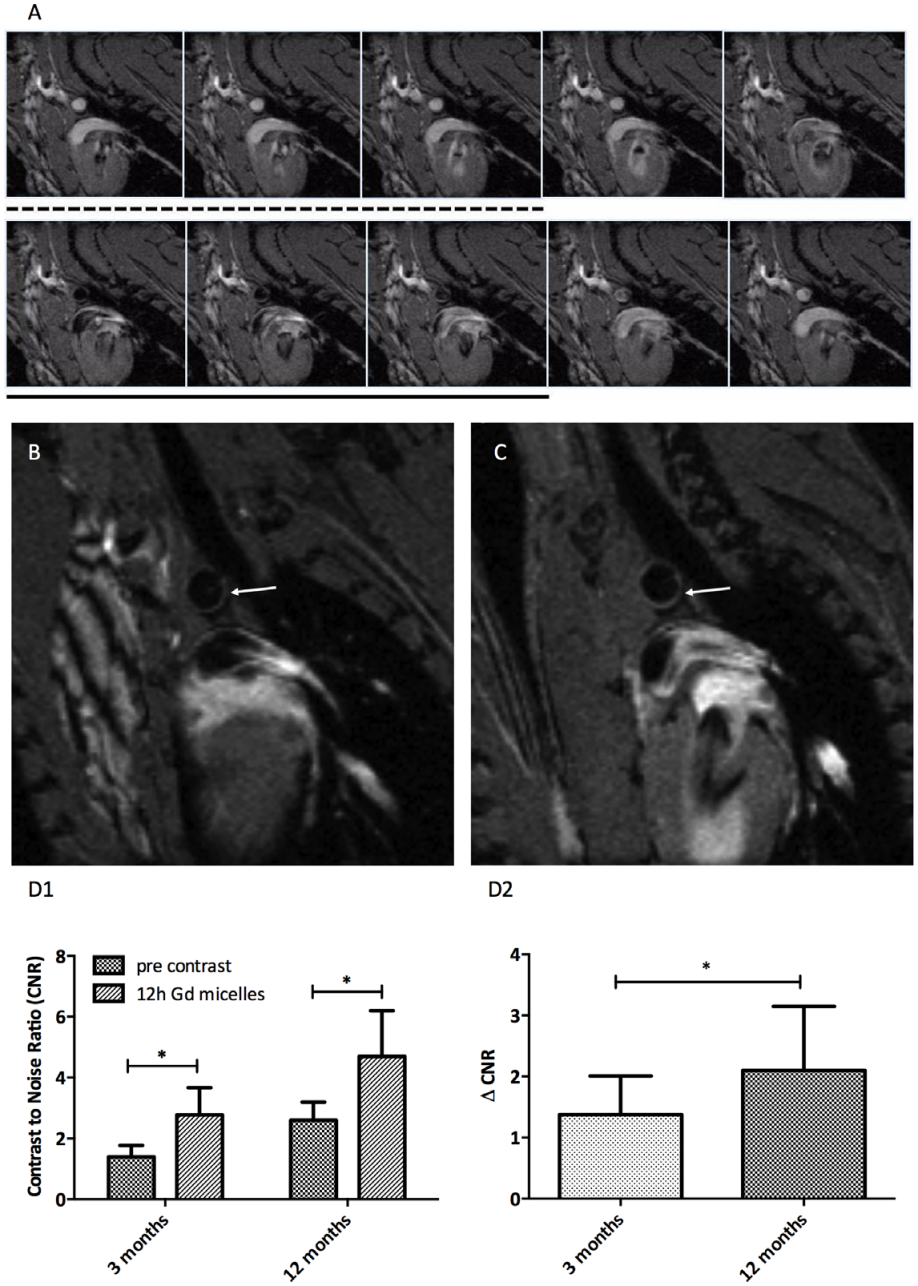
Atherosclerotic plaque detection in a cross-section of the aortic arch, including the effect of Gd-loaded micelles. A. Ten movie frames of a cross section of the aortic arch are generated. The black blood images used for positive contrast agent detection in the aortic arch are typically as those in image 6–8 (underlined). Circles indicate the region of the aortic arch cross section. White blood images 1–3 (dashed line), were used for the analysis of negative contrast agents B. A cross section of the aortic arch is shown before injection of micelles. Presumptive plaque regions are difficult to discriminate (arrow). C. Cross section of the aortic arch 12 hours after injection of micelles shows contrast enhancement on the basis of the aortic arch (arrow) D. Contrast to Noise Ratio (D1) and delta CNR (D2) of atherosclerotic plaques on the inner curvature of the aortic arch in 3 months old and 12–14 months old ApoE^−/−^ mice on a western diet.

The aortic arches of ApoE^−/−^ mice (young and aged) were imaged with the retrospective-gated MRI protocol pre- and post-injection of a micellar T1 contrast agent. Throughout the cardiac cycle, 10 movie frames of 6 transversal slices were made (Figure 3A). Pre contrast agent injection, the plaque burden in the inner curvature of the aorta was difficult to discriminate from healthy vessel wall, with only slightly elevated CNR in the plaque (arrow in Figure 3B). After injection of Gd-containing micelles, a distinct hyperintensity was observed in the inner curvatures of the aortic arch and carotid arteries at the well-documented locations where atherosclerotic plaque is found in these ApoE^−/−^ mice (Figure 3C) [31]. The hyper enhancement was largest in the aged animals, but could be distinguished in the younger animals as well.

The CNR values for these mice were determined after micelle injection for the images in the cardiac cycle with black blood (Figure 3A, frames 6–8). Quantitative CNR values for the young and aged animals are summarized in Figure 3D. The CNR increased significantly after the injection of micelles in both groups, but both the CNR (Figure 3D1) and **Δ**CNR (Figure 3D2) of the aged animals were systematically higher than in the younger animals.

**Figure 4:**
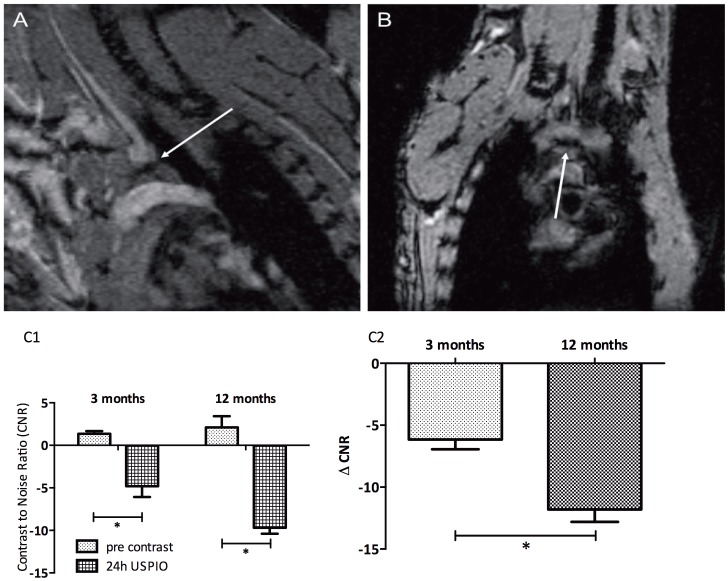
Detection of atherosclerotic lesions in the aortic arch using USPIOs. T2* effects of USPIO were observed on the basis of the aortic arch 24 hours after i.v. contrast agent injection. CNR significantly decreased from 2.1±1.3 before injection of contrast agent to −9.7±0.7, 24 hours after injection of micelles. The typical blooming effect by the USPIOs (arrow) was best observed in frontal views (B) of the aortic arch. C. CNR (C1) and delta CNR (C2) of both age groups before and 24 hours after USPIO injection.

We applied a similar imaging strategy to detect the contrast changes pre- and post-injection of an ultrasmall iron-oxide particle (USPIO). Because USPIOs induce large signal voids in the artery wall, the bright blood frames of the retrospective-gated CINE MRI were more suitable for CNR quantification (Figure 4A&B). CNR (Figure 4C1) and ΔCNR (Figure 4C2) values became negative (hypointense) after injection of the contrast agent, and as for the micelles, the enhancement was more pronounced in the aged mice.

### Age Related Differences in Vessel Wall Stiffness

**Figure 5:**
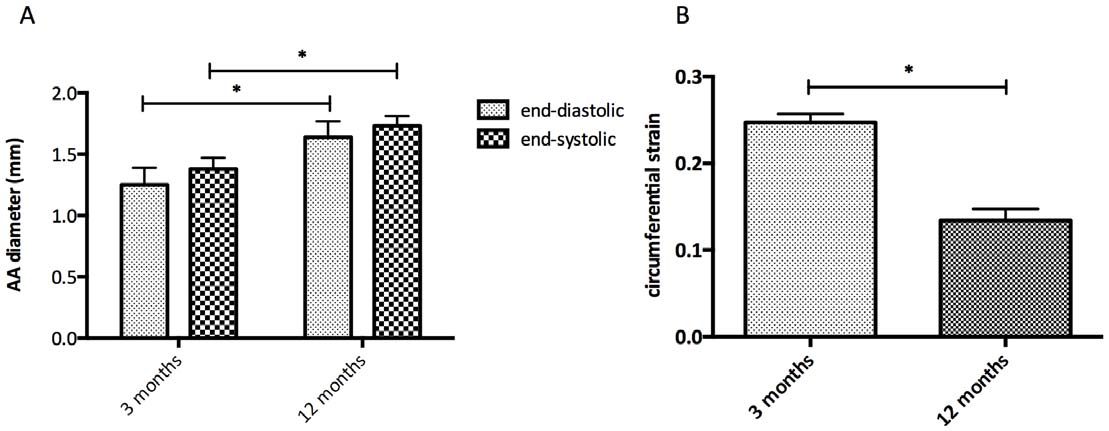
Vessel wall chracteristics measured by MRI. A. Diameter of the aortic arch in mm measured at end-diastole and end-systole measured in CINE MRI images from 3 months and 12 months old ApoE^−/−^ mice B. Distensibility of the aortic arch measured by the average maximal circumferential strain calculated for both age groups.

An increase in the diameter of the ascending aorta of the aged mice compared to the younger animals was observed both at end-systole and end-diastole (Figure 5A). A significant decrease of 46% in average maximal circumferential strain values was observed between 3 and 12-month-old ApoE^−/−^ mice (Figure 5B).

### Effect of Statin Treatment on Aortic Plaque and Vessel Stiffness

We assessed whether the effects of statin therapy could be observed both on plaque level as well as the stiffness of the aortic arch in ApoE^−/−^ mice.

**Figure 6:**
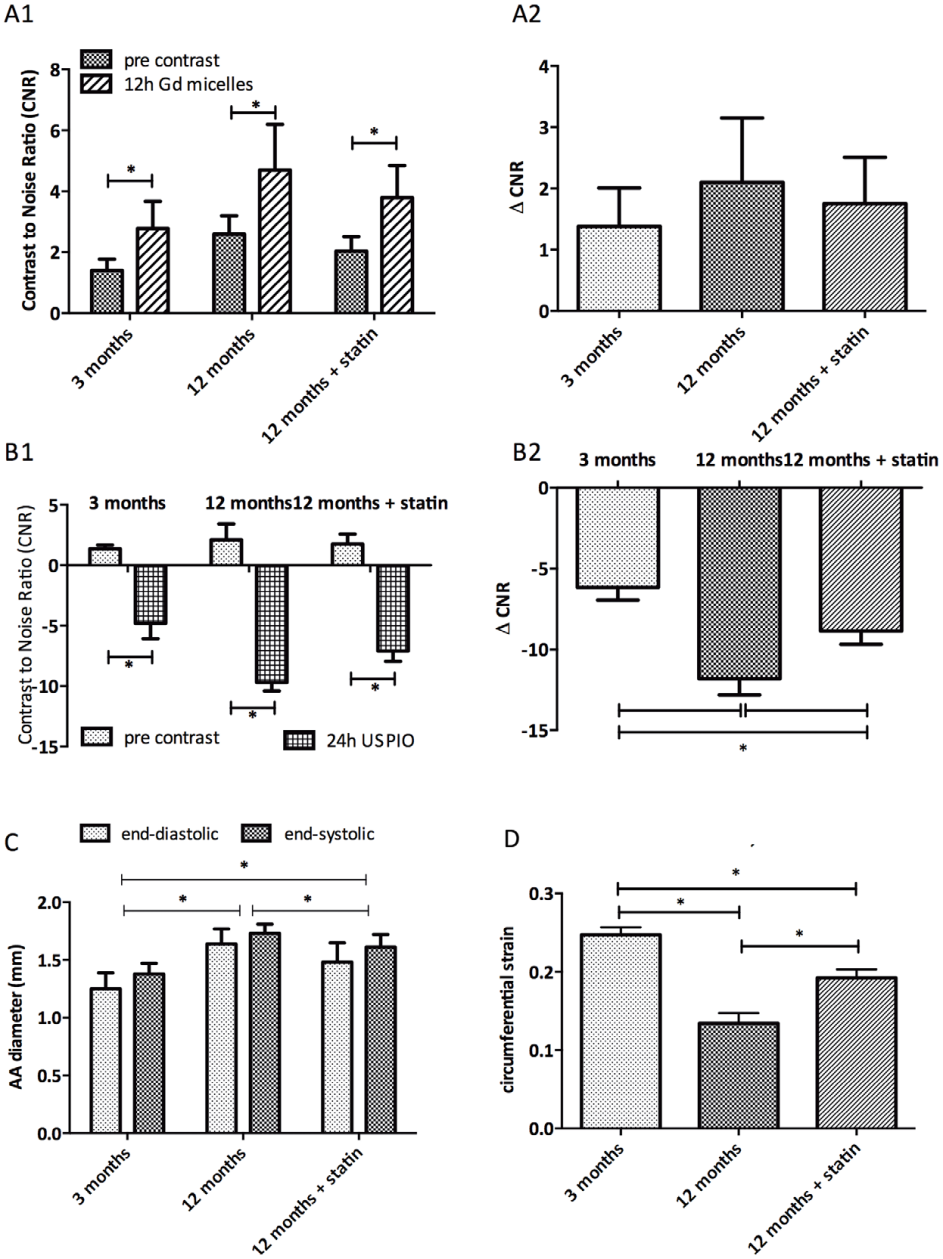
The effect of atorvastatin treatment on atherosclerotic plaques. A. CNR (A1) and ΔCNR (A2) of atherosclerotic plaques on the inner curvature of the aortic arch of 3 months old as well as 12–14 months old ApoE^−/−^ mice on a western diet with or without supplementation with atorvastatin after micelle injection. B. CNR (B1) and ΔCNR (B2) of atherosclerotic plaques on the inner curvature of the 3 treatment groups after USPIO injection. C. Diameter of the aortic arch in mm measured at end-diastole and end-systole measured in CINE MRI images in all 3 ApoE^−/−^ treatment groups. D. Average maximum circumferential strain values of the 3 treatment groups.

In aged ApoE^−/−^ mice treated for 12 weeks with a Western diet supplemented with atorvastatin plaques CNR changes were significantly smaller both in micelle injected mice (Figure 6A1 CNR, 6A2 ΔCNR) as well as USPIO injected mice (Figure 6B1 CNR, 6B2 ΔCNR) compared to age-matched untreated mice.

Diameter of the aortic arch in mm was measured at end-diastole and end-systole measured in all 3 ApoE^−/−^ treatment groups (Figure 6C). The diameter at both end-diastole and end-systole was significantly decreased in the 12-months-old statin-treated mice compared to the untreated 12-months-old animals, yet still significantly higher than in the young mice. The average maximal circumferential strain calculated during the cardiac cycle shows a decrease with age (Figure 6D). A statistically significant lower decrease in strain was noted after statin treatment.

### Correlation between MRI Contrast Enhancement, Aortic Stiffness and Histological Plaque Area

**Figure 7:**
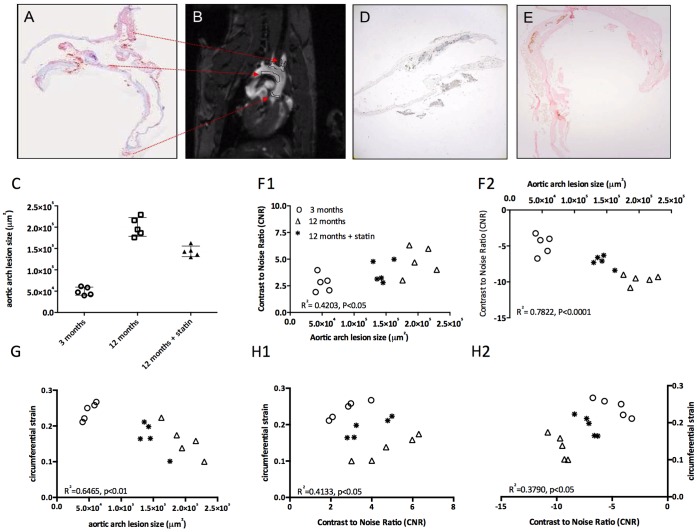
Histological validation of atherosclerosis and MRI. A. Lipid depositions on the basis of the aortic arch and in the branches to the carotid and brachiocephalic arteries were shown by Oil Red O staining. B. Regions with atherosclerotic plaques corresponding to the regions in A are depicted in this MR image of the aortic arch. C. Plaque sizes of the 3 treatment groups in µm2 determined on histological slices. D. Anti-Gd-DTPA immunohistochemical DAB staining localized the micelles in atherosclerotic plaques. E. Iron deposits are visualized with Prussian blue enhanced with DAB in the wall of the aortic arch. F. Correlation CNR of atherosclerotic plaques on the inner curvature of the aortic arch (F1 micelles, F2 USPIO) with plaque sizes of the 3 groups determined on histological slices. G. Correlation of the aortic arch lesion area with the circumferential strain of the 3 treatment groups. H. Correlation of the CNR of both micelles (H1) as well as USPIO (H2) with the circumferential strain for all data-points together.

The plaque area in the inner curvature of the aortic arch was independently determined using histology. The anatomical position of the plaques found on MR images corresponds to the areas observed by lipid staining with Oil Red O (Figure 7A & 7B). The plaque lesion sizes for the different groups are shown in Figure 7C. The plaque size was smallest for the young animals, largest for the aged animals. After statin treatment, the plaque size was reduced by 28.5%, but still significantly larger than in young ApoE^−/−^ mice. Using an anti-Gd-DTPA staining, the presence of micelles could be detected in distinct plaque areas: at the inner curvature of the aortic arch and in the intima of the vessel wall, in line with our MRI results. In contrast, the hypointense plaque regions due to iron-oxide accumulations mainly occurred in the plaque core, which was confirmed by Prussian Blue staining (Figure 7D & 7E).

Contrast enhancement on MRI for individual animals correlated very well (p<0.01) with the lesion size as determined with histology (Figure 7F1 micelles, F2 USPIOs), also after statin treatment, with an R^2^ of 0.4203 and 0.7822 for the Gd-micelles and the USPIO respectively.

The circumferential strain showed a linear correlation with plaque size, as determined by histology (Figure 7G), with an R^2^ of 0.6465 (p<0.01) respectively. Lastly, linear regression showed that the circumferential strain was also correlated to the CNR after contrast agent administration (Figure 7H1 micelles, R^2^ 0.4133 p<0.05 and 7H2 USPIO R^2^ 0.3790, p<0.05).

## Discussion

### Self-Gated CINE MRI for Atherosclerotic Plaque Detection

Atherosclerotic plaque formation typically originates in the aortic root and progresses to the ascending aorta, the aortic arch and onward through the aorta’s principal branches leading to progressive arterial stiffness [4]. These anatomical positions have one common theme; low and oscillatory (multi- or bidirectional) flow patterns, implying that plaque detection may be hampered by alterations in blood flow [32].

Proper visualization and quantification of atherosclerotic plaque components in both patients and animal models usually relies heavily on black-blood or bright-blood techniques with saturation slices or double inversion recovery methods [33], [34]. However, the required steady state blood saturation can be difficult to maintain in ECG-triggered sequences [18] especially in animal models. In the aortic arch, the prime site of plaque development, and carotid arteries assessment of plaques and vessel wall area becomes even more difficult, because of its proximity to the beating heart which may cause large motion artifacts on top of flow artifacts.

Classically, synchronization with the heart cycle, or prospective gating, is done using respiratory and ECG sensors to generate triggering signals [35]. In hemodynamically unstable animals, one needs to monitor the R-R interval closely, or choose this interval conservatively, which means that the total scan time will be longer and as a consequence the influence of the imaging session on animal welfare will be higher.

Therefore, the use of a retrospective-gated CINE MRI sequence provides distinct advantages over a triggered CINE or single-frame sequence. Firstly, the maintenance of steady state saturation of the retrospective-gated sequence helped to reduce in-flow artifacts. Secondly, the retrospective-gated CINE sequence covered the full cardiac cycle without dead time at the end of the cardiac cycle waiting for the next ECG trigger. Using the retrospective-gated CINE sequence in combination with contrast agents known to accumulate in atherosclerotic plaques, micelles as well as USPIO, allowed for a good discrimination of the atherosclerotic lesion on the inner curvature of the aortic arch [9], [10].

### Plaque Burden and Aortic Stiffness

The finding of age-related increases in aortic stiffness, vessel diameter and aortic atherosclerosis is consistent with prior studies [36]. Traditionally, the stiffness and compliance of conduit vessels is an estimate from the pulse wave velocity (PWV) [37], [38]. This is routinely done in the clinic using ultrasound or MRI, yet PWV measurements in mice are feasible as shown in the group of Jakob, but challenging due to the high heart rate and difficulties to measure flow velocities *in vivo* [15], [16]. Using the stiffness of the vessel wall can be a good alternative or additional tool to characterize vessel compliance. The correlation between aortic stiffness and plaque burden is particularly interesting because the elastic properties of the aortic wall play an important role in the pathogenesis of cardiovascular disease including atherosclerosis and hypertension, and is an independent risk factor for ventricular hypertrophy and stroke. Further pathophysiological studies may include longitudinal follow-up experiments to assess the temporal relationship between vascular compliance and plaque burden as well as the ageing related increase in vessel wall diameter.

Our study showed that in a ApoE^−/−^ mouse model the histological plaque burden was closely related to both contrast-enhancement on MRI and the aortic distensibility. This correlation was preserved over a large age range, also during statin treatment, indicating that in this mouse model, changes in aortic stiffness are dominated by the plaque burden. The average circumferential strain decreases with age, whereas statin treatment slows down this decrease. Together with the observation of an increase in vessel wall diameter this points at a decrease in arterial elasticity and compliance with age and in relation to the extent of atherosclerosis progression.

Therefore, aortic compliance measurements may be an alternative approach in this animal model to monitor subtle changes of the arterial wall elasticity to actual plaque imaging, which is still difficult and time-consuming. Aortic circumferential strain measurements would provide a straight-forward method to study the response to various dietary and pharmacological manipulations both in this animal models and patients [39], [40]. Though we found a very clear correlation between aortic stiffness and plaque burden in the ApoE^−/−^ mouse, this is not necessarily the case in other mouse models, nor in patients. Arterial stiffness is an independent predictor of ventricular hypertrophy and stroke in patients, indicating that other mechanisms than plaque development may cause stiffening of the arteries. Assessing the temporal relationship between vascular compliance and plaque burden, may be particularly useful in different mouse models of atherosclerosis, including models of vascular dysfunction.

### Conclusion

We have shown that retrospectively gated CINE MRI can be used to detect plaque burden and aortic distensibility simultaneously. Because the method can be used for both black-blood and bright-blood contrast, it is suitable for both gadolinium- and iron oxide based contrast agents. We have shown that in the ApoE^−/−^ mouse there is a high correlation between aortic stiffness, and plaque load, and both measures can be used to assess atherosclerotic plaque progression and therapeutic interventions.

## Supporting Information

Figure S1 **Time course of micelles and USPIO. A.** Time course of Gd-micelle accumulation in the inner curvature of the aortic arch of ApoE^−/−^ mice. Contrast to Noise Ratios (CNR) were determined at different time points after intravenous injection of n = 8 mice. **B.** CNR determined at different time points after USPIO injection in the inner curvature of the aortic arch of n = 8 mice.
(TIF)

Figure S2 **Diameter measurements with different numbers of movie frames.** Aortic arch diameter measurements at end-systole and end-diastole for 5, 8, 12, 15, 20 and 40 reconstructed cardiac movie frames compared to 10 movie frames. *P<0.05 compared to 10 movie frames.
(TIF)

Figure S3 **Anatomical positioning in the aortic arch. A.** Depiction of the position (in green) in the aortic where frames were taken orthogonal to the aortic arch. **B.** Schematical depiction of determination of the diameter of the aortic arch using circular cross-sections only.
(TIF)
