# Supplementary figures and images for: Self-Gated CINE MRI for Combined Contrast-Enhanced Imaging and Wall-Stiffness Measurements of Murine Aortic Atherosclerotic Lesions
Source: PLoS One. 2013 Mar 5;8(3):e57299. doi: 10.1371/journal.pone.0057299 (PMC3589480; doi:10.1371/journal.pone.0057299)

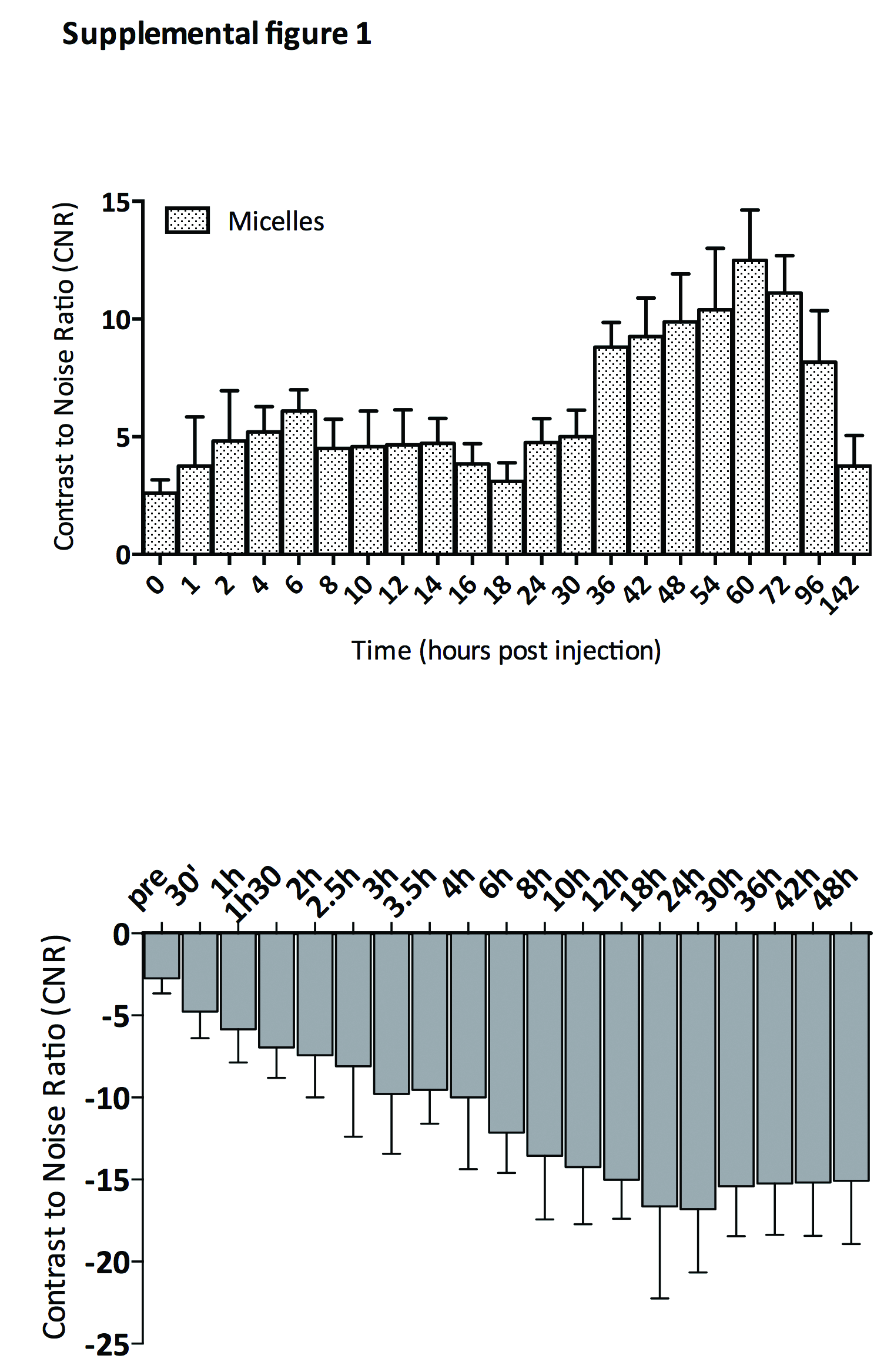

Supplement: Figure S1 — Time course of micelles and USPIO. A. Time course of Gd-micelle accumulation in the inner curvature of the aortic arch of ApoE−/− mice. Contrast to Noise Ratios (CNR) were determined at different time points after intravenous injection of n = 8 mice. B. CNR determined at different time points after USPIO injection in the inner curvature of the aortic arch of n = 8 mice. (TIF) [file pone.0057299.s001.tif]

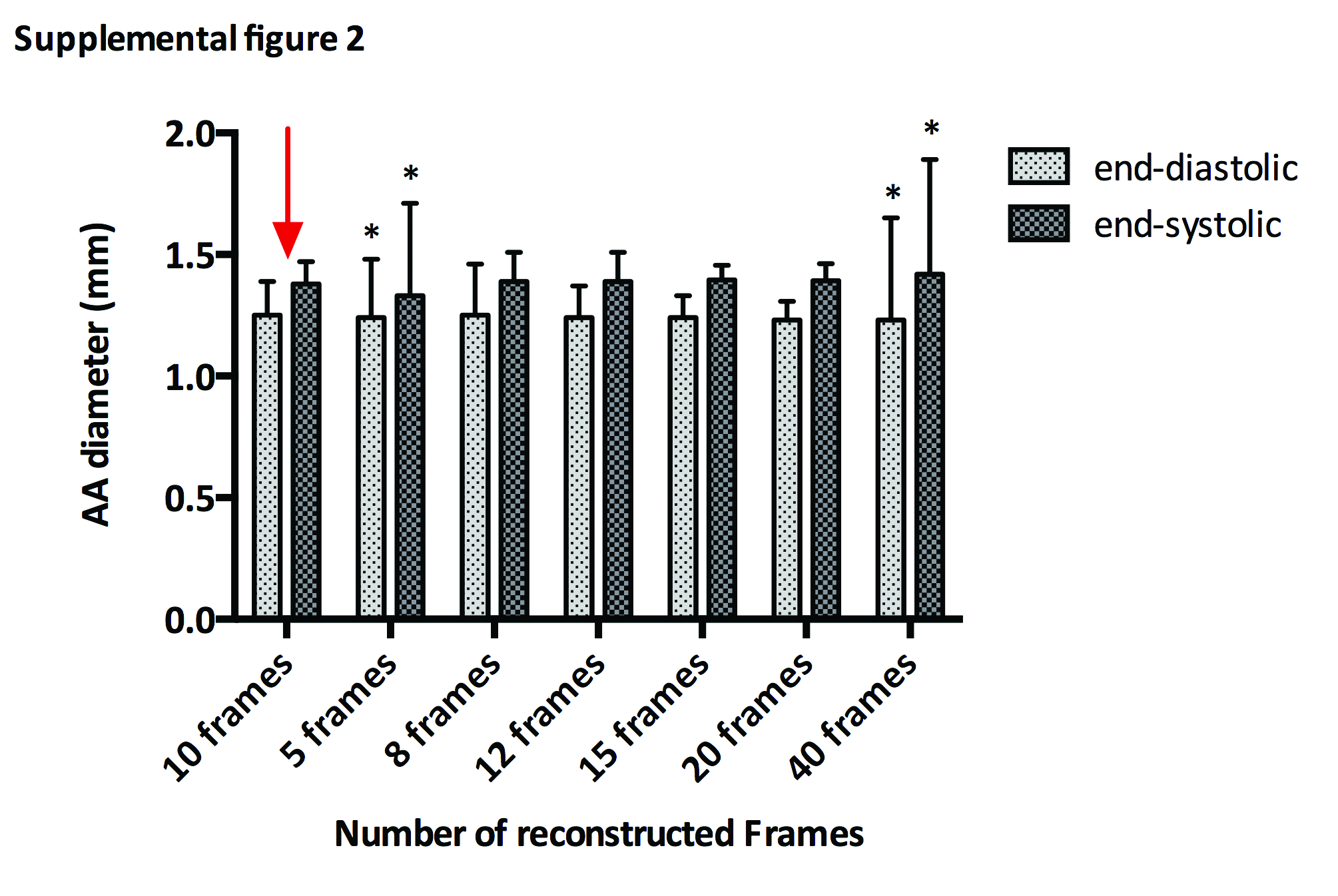

Supplement: Figure S2 — Diameter measurements with different numbers of movie frames. Aortic arch diameter measurements at end-systole and end-diastole for 5, 8, 12, 15, 20 and 40 reconstructed cardiac movie frames compared to 10 movie frames. *P<0.05 compared to 10 movie frames. (TIF) [file pone.0057299.s002.tif]

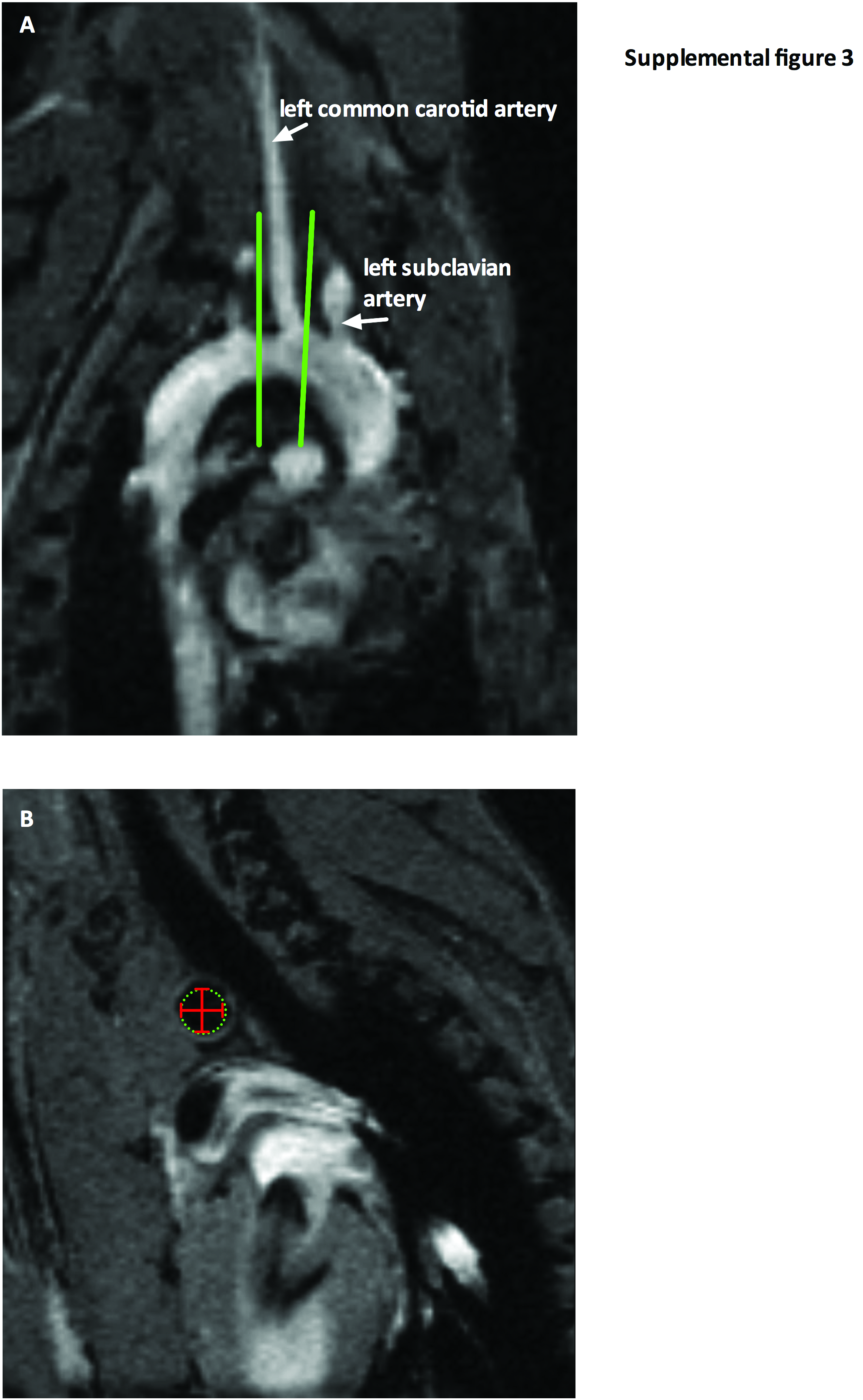

Supplement: Figure S3 — Anatomical positioning in the aortic arch. A. Depiction of the position (in green) in the aortic where frames were taken orthogonal to the aortic arch. B. Schematical depiction of determination of the diameter of the aortic arch using circular cross-sections only. (TIF) [file pone.0057299.s003.tif]
